# Surgical Complications in Hirschsprung Disease and the Impact of Botulinum Toxin Injection on Hirschsprung-Associated Enterocolitis

**DOI:** 10.3390/jcm15041665

**Published:** 2026-02-23

**Authors:** Fatma Özcan Siki, Mehmet Sarikaya, İlhan Çiftci, Gamze Kaygisiz Bayindir, Metin Gündüz, Tamer Sekmenli

**Affiliations:** Department of Pediatric Surgery, Faculty of Medicine, Selcuk University, Konya 42900, Turkey; mehmet.sarikaya@selcuk.edu.tr (M.S.); driciftci@selcuk.edu.tr (İ.Ç.); gamzekaygisizbayindir@selcuk.edu.tr (G.K.B.); tamersekmenli@selcuk.edu.tr (T.S.)

**Keywords:** Hirschsprung disease, Hirschsprung-associated enterocolitis, botulinum toxin injection, long-segment Hirschsprung

## Abstract

**Background:** Hirschsprung disease (HD) is associated with substantial postoperative morbidity, particularly due to Hirschsprung-associated enterocolitis (HAEC), despite definitive surgical treatment. Postoperative outcomes may vary according to the length of the aganglionic segment and the surgical technique used, and optimal management of recurrent HAEC remains a significant clinical challenge. **Methods:** The medical records of patients who underwent corrective surgery for HD between 2011 and 2023 were retrospectively reviewed. Demographic characteristics, disease segment length, surgical technique, postoperative complications, and HAEC episodes were recorded. HAEC diagnosis and follow-up assessments were conducted in accordance with the Delphi criteria. Patients with recurrent HAEC and obstructive symptoms refractory to standard conservative management were evaluated for botulinum toxin injection (BTI). **Results:** A total of 72 patients were included in the study. The majority of the patients were male (83.3%), with a mean age at diagnosis of 11 months. The Duhamel procedure was the most frequently performed surgical technique. Although the rate of anastomotic leakage was greater in patients who underwent the Swenson procedure compared with the Duhamel group, this difference did not reach statistical significance when the appropriate statistical methods were used because the small sample size. No significant difference in the incidence of HAEC was observed among the different surgical techniques. BTI was administered to 13 patients, and regression of enterocolitis episodes according to the Delphi criteria was observed in 11 patients (84.6%). Most postoperative complications are observed in patients with long-segment Hirschsprung disease. **Conclusions:** Postoperative complications and HAEC remain major clinical challenges in the management of Hirschsprung disease, particularly in patients with long-segment involvement. Although the surgical technique may influence certain complication rates, HAEC appears to be a multifactorial condition. Botulinum toxin injection may serve as a supportive treatment option in selected patients with refractory HAEC; however, prospective controlled studies are needed to further clarify its role.

## 1. Introduction

Hirschsprung’s disease (HD) is a developmental disorder of the enteric nervous system characterized by the absence of ganglion cells in the distal intestine. The embryological basis of this disease lies in the incomplete craniocaudal migration of neural crest cells during embryonic development. This migration defect results in the failure of the formation of the myenteric and submucosal plexuses, leading to a permanent functional intestinal obstruction in the affected intestinal segment. The length of aganglionosis can vary, resulting in a wide clinical spectrum from short-segment disease to total colonic aganglionosis [1,2].

The pathophysiology of HD is not limited to the absence of ganglion cells but rather involves complex interactions among the enteric nervous system, smooth muscle cells, interstitial Cajal cells, and the mucosal immune response. Genetic studies have demonstrated that multiple genetic mutations—most notably those involving the RET proto-oncogene—contribute to development. This genetic heterogeneity is considered a significant factor in explaining both the phenotypic diversity of the disease and the differences in postoperative functional outcomes [3,4].

Clinically, HD often presents in the neonatal period with delayed meconium passage, abdominal distension, and vomiting; however, in some cases, a diagnosis may be made at a later age. In patients with delayed diagnosis, recurrent episodes of constipation, enterocolitis, and feeding problems become more pronounced. Delayed diagnosis increases the risk of preoperative complications and negatively impacts long-term morbidity [2,5,6].

The primary treatment for HD involves surgical approaches consisting of the resection of the aganglionic segment and the pull-through of the ganglionated bowel to the anus. Although surgical morbidity has decreased with the development of minimally invasive and transanal approaches, with the Swenson, Duhamel, and Soave procedures being the most widely used techniques today, postoperative complications remain a significant concern. The choice of surgical technique varies depending on the segment length of the disease, the surgeon’s experience, and patient-specific anatomical features; however, it is reported that no single method eliminates complications [2,7,8].

One of the most severe and potentially life-threatening complications encountered in the postoperative period is Hirschsprung-associated enterocolitis (HAEC). HAEC can manifest both preoperatively and postoperatively, with a reported incidence in the literature ranging from approximately 15% to 50%. The clinical presentation spans a broad spectrum, from mild abdominal distension and diarrhea to toxic megacolon and sepsis. The etiology of HAEC has not been fully elucidated; however, it is thought that multiple mechanisms, including intestinal dysmotility, mucosal barrier dysfunction, and immune dysregulation, are thought to play a role [9,10].

HAEC is not universally accepted for its prevention and management of HAEC. In recent years, attention has been given to the role of internal anal sphincter hypertonicity in the pathogenesis of HAEC, leading to the emergence of botulinum toxin injection (BTI) as a nonsurgical supportive treatment option. Botulinum toxin reduces sphincter tone and facilitates distal intestinal passage by inhibiting acetylcholine release. Current studies indicate that BTI may reduce enterocolitis episodes and effectively control symptoms in certain patient groups; however, these data are supported by a limited number of studies [2,11,12].

This study aimed to evaluate the spectrum of postoperative complications, the incidence of HAEC, and the therapeutic impact of BTI on HAEC in patients who underwent surgery for Hirschsprung’s disease. Specifically, this study is intended to elucidate the role of BTI as a supportive adjunct option in clinical practice.

## 2. Materials and Methods

### 2.1. Study Design and Ethical Approval

**Figure 1:**
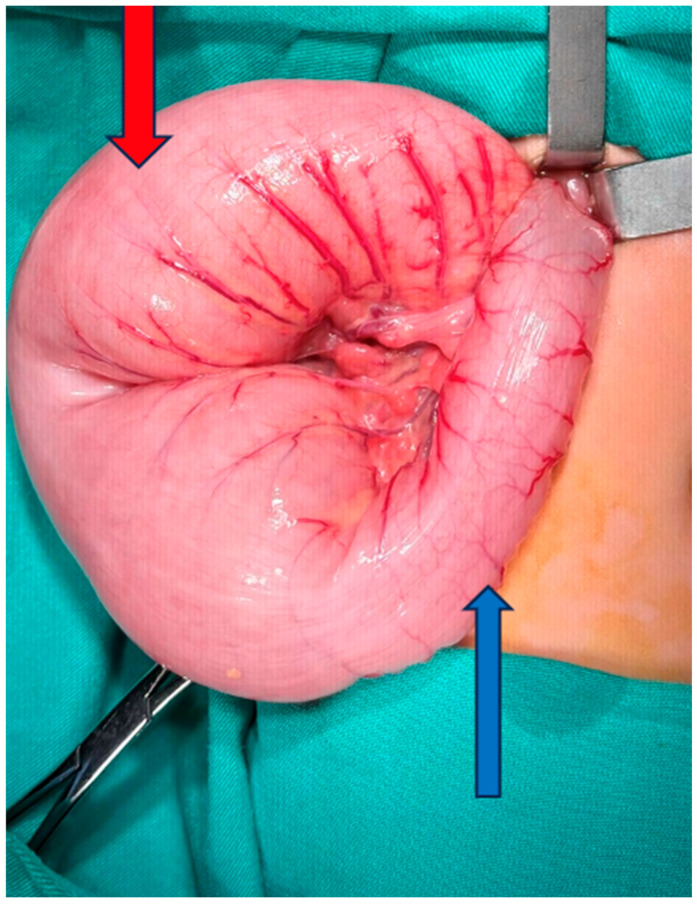
Perioperative image of ganglionic and aganglionic segments. Blue arrow: aganglionic segment. Red arrow: ganglionic segment.

This study was conducted through a retrospective review of the medical records of patients who underwent corrective surgery for HD at our clinic between 2011 and 2023 (Figure 1). The study was approved by the Ethics Committee of the University (Approval No: 327, Date: 2025). Due to the retrospective design, individual informed consent was not needed.

### 2.2. Patient Selection and Eligibility Criteria

Histopathological confirmation of HD diagnosis and availability of adequate postoperative follow-up data were established as inclusion criteria, while patients with incomplete clinical data were excluded from the study.

### 2.3. Data Collection and Clinical Variables

Patient demographics, disease segment length (short-segment, long-segment, and total colonic aganglionosis), associated syndromes and anomalies, age at diagnosis and age at surgery, surgical techniques performed, occurrence of anastomotic leakage in the early postoperative period, presence of HAEC, anastomotic dilatation procedures, and data regarding BTI administered following recurrent HAEC episodes were recorded. Additionally, cases of late ileus presenting with obstructive symptoms developing three months after corrective surgery and cases of adhesive ileus requiring surgical intervention were evaluated. Anastomotic stricture was defined as a condition leading to clinical symptoms that required mechanical or endoscopic dilatation.

### 2.4. Diagnosis and Grading of HAEC

**Figure 2:**
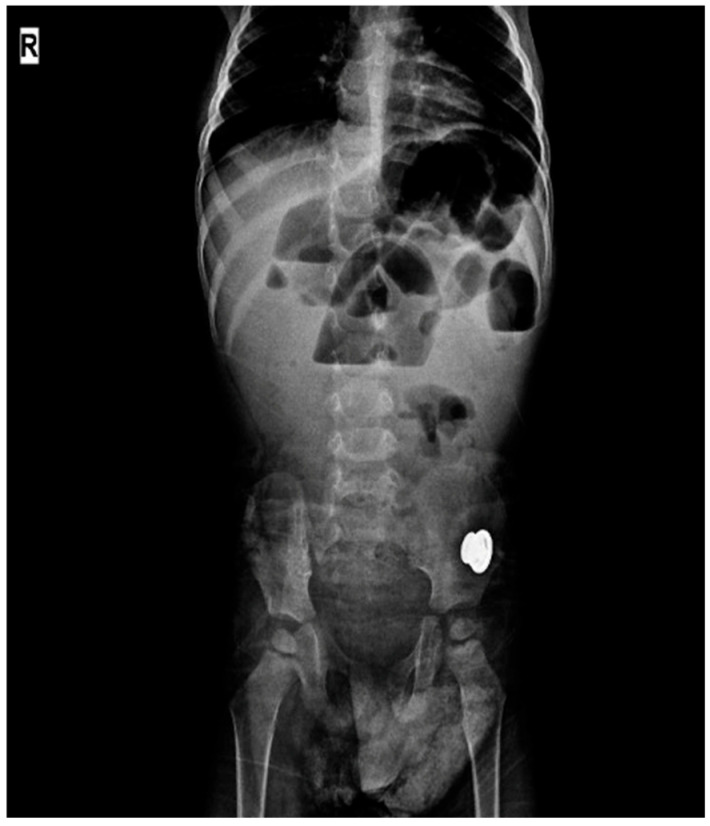
Abdominal X-ray image of a patient presenting with Hirschsprung-associated enterocolitis (HAEC). Disseminated air-fluid levels are present.

To minimize the diagnostic heterogeneity reported in the literature, the diagnosis of HAEC was established via the Delphi consensus criteria. These criteria are based on a multicomponent assessment system that includes clinical findings, physical examinations, radiological evaluations, laboratory and pathological findings, and treatment requirements. All HAEC diagnoses and enterocolitis assessments during follow-up after BTI were performed in accordance with the Delphi criteria (Figure 2). A detailed schema and definitions of these criteria are presented in Table 1 [9,10].

### 2.5. Indications for Botulinum Toxin Injection

In the postoperative period following definitive surgery, patients who developed recurrent HAEC episodes accompanied by obstructive clinical findings, despite several weeks of standard conservative management, including antibiotic therapy, regular rectal irrigation, and dietary modification, were evaluated for BTI. These patients belonged to the moderate-to-severe HAEC group (Grades II–III) according to the Delphi criteria and represented patients with persistent obstructive symptoms refractory to conservative treatment [13].

**Figure 3:**
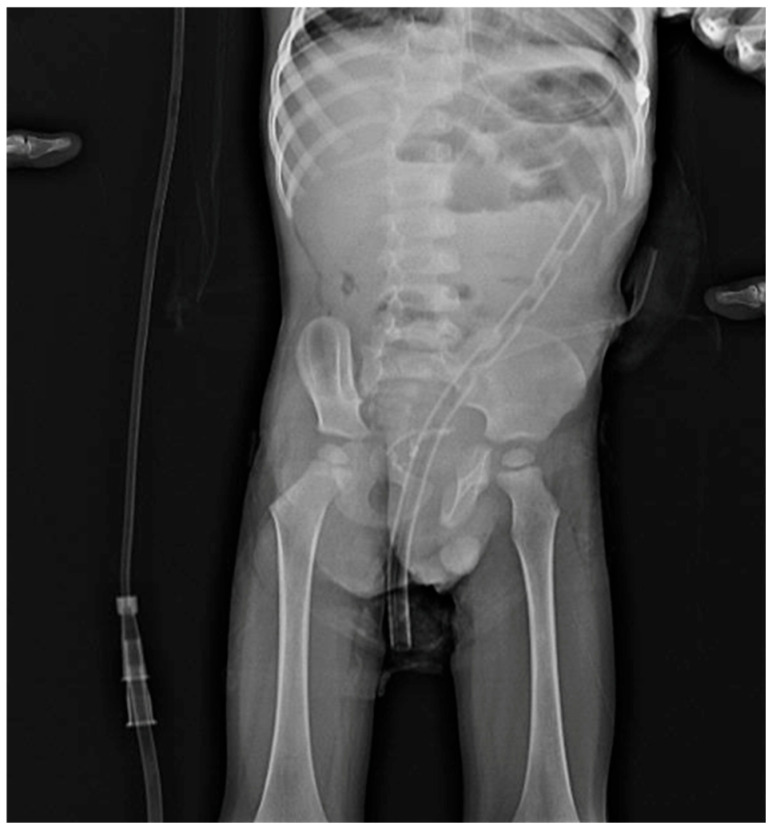
Abdominal X-ray showing the rectal tube and nasogastric catheter in a patient with Hirschsprung-associated enterocolitis (HAEC).

In this context, BTI was used not in mild HAEC cases but rather as a supportive intermediate treatment option prior to surgical intervention in patients with prolonged or recurrent episodes who failed to show clinical improvement despite several weeks of appropriate conservative therapy (Figure 3).

### 2.6. Botulinum Toxin Injection Procedure

The BTI procedure was performed under general anesthesia. During the application, injections were administered into the internal anal sphincter in four quadrants (at the 3, 6, 9, and 12 o’clock positions) using a 25-gauge needle. This approach was preferred to ensure uniform homogeneous relaxation of the internal anal sphincter [2,11,14].

The Botulinum toxin dosage was determined as 4–6 units/kg, which is consistent with safe and effective applications reported in children with Hirschsprung disease. Botulinum toxin type A was diluted with 1 mL of physiological saline and injected into the internal anal sphincter using palpation [15]. All injections were performed by a single pediatric surgeon. Patients were clinically evaluated at the 2nd week, 1st month, and 3rd month post-procedure; in cases where obstructive symptoms or HAEC episodes, as defined by the Delphi criteria, recurred, the BTI application was repeated [11,12,16]. In the evaluation conducted through patient records, the presence or absence of temporary or permanent adverse events related to BTI (temporary incontinence, bleeding, infection, or systemic toxicity) was documented.

### 2.7. Statistical Analysis

Continuous variables are expressed as the mean ± standard deviation or median (minimum–maximum), whereas categorical variables are presented as numbers and percentages (%). The normality of the data distribution was assessed using the Shapiro–Wilk test. Depending on the distribution characteristics, Student’s *t*-test or Mann–Whitney U test was utilized for the comparison of continuous variables. For the analysis of categorical variables, the Pearson chi-square test or Fisher’s exact test was applied based on the expected cell counts. A *p*-value of <0.05 was considered statistically significant. All statistical analyses were performed using IBM SPSS Statistics version 25.0 (IBM Corp., Armonk, NY, USA).

## 3. Results

### 3.1. Patient Characteristics and Demographics

Of a total of 74 patients treated for HD at our clinic, 1 child who was lost to follow-up before definitive surgery and 1 patient who died following cardiac surgery before corrective surgery could be performed were excluded. Thus, a total of 72 patients were included in the final analysis. Twelve patients were female (16.7%), and sixty were male (83.3%). The mean age at diagnosis was found to be 11 months (range: 2 days–13 years). In patients diagnosed with total colonic aganglionosis, the mean age at diagnosis was 3 ± 0.5 days. The mean age at which corrective surgery was performed was determined to be 23 months (range: 8 months–14 years).

Total colonic aganglionosis was identified in eight patients, short-segment HD in 28 patients, and long-segment HD in 36 patients.

### 3.2. Associated Anomalies and Comorbidities

Ten patients had a diagnosis of Down syndrome, and eight of them had concomitant immunodeficiency. One patient was diagnosed with Crohn’s disease following further investigation due to failure to thrive during follow-up. Coexistence of HD and anorectal malformation (high-type atresia without fistula) was observed in two patients. In one patient, HD was diagnosed via biopsy during the neonatal period due to a megacolon appearance accompanied by duodenal atresia and rotational anomaly.

### 3.3. Surgical Techniques and Postoperative Interventions

Regarding the surgical techniques performed, 56 patients underwent the Duhamel procedure, 8 patients underwent the Swenson procedure, and 8 patients underwent transanal endorectal pull-through (TEPT). Following corrective surgery, 12 patients required dilatation due to stricture at the anastomotic line; six of these patients underwent repeated dilatation procedures.

Due to late complications, three patients with a diagnosis of intestinal obstruction underwent surgical intervention. Detailed data regarding the number of patients, associated anomalies and syndromes, surgical techniques performed, and postoperative complications are presented in Table 2.

### 3.4. HAEC and Botulinum Toxin Injection Outcomes

A total of 13 patients received BTI. Among these patients, five patients received BTI twice, one patient three times, and one patient four times. The mean interval between BTI applications was recorded as 4.3 months. During the study period, HAEC episodes were observed in 14 patients; while five of these patients did not receive BTI, nine were in the group that received BTI. After a single BTI application, a regression in enterocolitis episodes was detected in 6 of the patients who received BTI. Long-segment HD was present in 9 of the patients who underwent BTI.

### 3.5. Outcomes According to Surgical Technique

**Figure 4:**
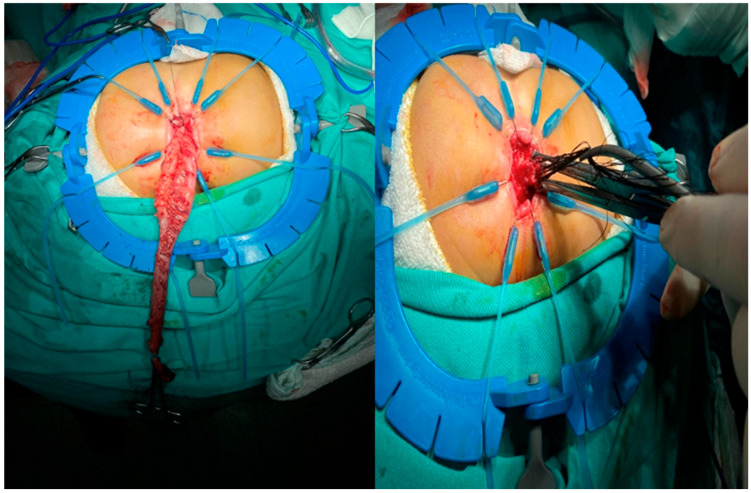
TEPT Technique: Removal of the transanal endorectal aganglionic segment and anastomosis with the ganglionic segment.

When the outcomes were evaluated according to surgical technique, the Duhamel procedure was the most frequently performed method (77.7%), followed by the Swenson and TEPT procedures (Figure 4). Anastomotic leakage was observed in 25% of the patients who underwent the Swenson procedure, whereas this rate was 8.9% in the Duhamel group. However, owing to the limited number of patients in the Swenson group, this difference did not reach statistical significance according to Fisher’s exact test (*p* = 0.209). Therefore, no definitive conclusions can be drawn regarding differences in anastomotic leakage rates among the surgical techniques. Anastomotic stricture was observed in 17.9% of the Duhamel group and 25% of the Swenson group, with no significant difference found between the groups. HAEC episodes were observed in 21.4% of patients in the Duhamel group and 25% of patients in the Swenson group; this difference was not statistically significant (*p* = 0.785). No postoperative complications were observed in the eight patients who underwent TEPT.

BTI was applied exclusively in patients who underwent the Duhamel procedure, accounting for 23.2% of this group. In 11 of the 13 patients (84.6%) who received BTI, a regression of enterocolitis episodes was recorded according to the evaluation made using the Delphi criteria. Furthermore, it was determined that the majority (78.2%) of the patients who developed complications were diagnosed with long-segment HD. Surgical outcomes, complication rates, and BTI application data according to surgical technique are presented in Table 3.

## 4. Discussion

In this study, the postoperative complications, incidence of HAEC, and clinical outcomes of BTI in patients undergoing surgery for HD were retrospectively evaluated. Our findings indicate that the choice of surgical technique influences certain complications, HAEC remains a significant cause of morbidity in the postoperative period, and BTI may be considered a supportive treatment option in a selected patient group.

Age at diagnosis and the duration until surgery are among the decisive factors for the clinical course and postoperative outcomes of HD. In our study, the mean age at diagnosis was 11 months, and it was observed that patients diagnosed with total colonic aganglionosis were diagnosed significantly earlier. This finding is consistent with the literature reporting that total colonic aganglionosis is diagnosed early because it typically presents with a more severe clinical picture during the neonatal period [5,17]. Conversely, a longer history of constipation and an increased risk of complications are reported in patients with a late diagnosis [18].

HD is a complex clinical entity that extends beyond being an isolated intestinal motility disorder, often presenting in association with various congenital anomalies and syndromes. In our study, concomitant anomalies such as Down syndrome, immune deficiency, and anorectal malformations were identified; notably, the coexistence of high-type anal atresia and Hirschsprung’s disease in two patients was notable. The literature indicates that while the association between anorectal malformation and Hirschsprung’s disease is rare, it is a well-defined condition in which diagnosis may be delayed, and surgical management can become more complicated [19,20,21].

Similarly, cardiac anomalies and genetic syndromes accompanying HD are observed more frequently, particularly in the presence of Down syndrome, and increased postoperative morbidity has been reported in these patients [22,23]. In our study, the presence of immune deficiency in a significant portion of patients diagnosed with Down syndrome suggests an increased predisposition to infections and enterocolitis episodes in this group. These findings support the notion that the development of HAEC is related not only to the surgical technique or intestinal segment length but also to patient-specific systemic factors.

The presence of associated anomalies can directly influence surgical timing, postoperative monitoring strategies, and the risk of complications. Our findings reveal that focusing solely on intestinal pathology is insufficient in the management of HD; a multidisciplinary approach is essential, especially in the presence of anorectal malformations, cardiac anomalies, and genetic syndromes. Individualizing the diagnostic and therapeutic process in this patient group is of critically importance for reducing postoperative complications and the risk of HAEC.

When evaluated in terms of surgical timing, corrective surgery was performed at a mean age of 23 months in our study. Although it has been suggested in the literature that performing surgery during the neonatal or early infantile period may provide certain functional advantages, it has been reported that surgical timing alone does not determine long-term outcomes; rather, disease segment length and associated anomalies are more decisive [2]. Our findings suggest that despite the wide range in surgical age, complications are more closely related to the segment length of the disease.

The most frequently performed surgical technique in our study was the Duhamel procedure, followed by the Swenson and TEPT techniques. The rate of anastomotic leakage was higher in patients who underwent the Swenson procedure compared with those treated with the Duhamel technique. This finding may be attributed to the more extensive pelvic dissection, increased tension at the anastomotic site, and technical challenges related to distal rectal mobilization inherent to the Swenson procedure. Previous studies have similarly reported that these technical characteristics may predispose patients undergoing the Swenson procedure to a higher risk of anastomotic leakage [8,24,25]. Although this difference did not reach statistical significance in the present study, likely due to the limited sample size, the observed trend represents a clinically relevant consideration when evaluating complication profiles associated with different surgical techniques. On the other hand, the absence of a significant difference in anastomotic stricture rates between surgical techniques suggests that this complication may be more closely related to postoperative healing dynamics, biological anastomotic maturation, and patient-specific factors rather than the surgical approach itself.

Hirschsprung-associated enterocolitis remained a significant cause of postoperative morbidity in our study. The lack of a significant difference in the incidence of HAEC between the Duhamel and Swenson groups is consistent with studies in the literature reporting that surgical technique alone does not determine the development of HAEC [9]. The multifactorial pathogenesis of HAEC involves intestinal motility disorders, mucosal barrier dysfunction, and immune response abnormalities [26].

The use of the Delphi criteria for the diagnosis of HAEC in this study is a significant methodological advantage that enhances the comparability of the results with the literature. Adopting a multi-component approach based on clinical, radiological, and treatment requirements in the definition of HAEC reduces diagnostic heterogeneity and ensures a more accurate identification of true HAEC cases [9,10]. Therefore, the HAEC rates reported in our study are considered to be more reliable.

BTI was administered to a selected group of patients in our study who presented with recurrent HAEC episodes and obstructive symptoms. The regression of enterocolitis episodes observed in the majority of patients receiving BTI, as defined by the Delphi criteria, is consistent with the clinical improvement findings reported in the literature [2]. However, as the patients receiving BTI represent a refractory subgroup, it is not possible to make a causal inference regarding this treatment method. Our findings suggest that BTI may be an effective approach for symptom control in the appropriate patient group.

It is noteworthy that the vast majority of patients who received BTI had long-segment Hirschsprung’s disease. It has been previously reported that the risk of postoperative complications and HAEC is higher in cases of long-segment and total colonic aganglionosis [9,16]. The fact that most of the patients who developed complications in our study were diagnosed with long-segment HD once again highlights the decisive role of disease segment length on the clinical course.

This study has several limitations. The retrospective design, limited sample size, and selection bias in the administration of BTI restrict the generalizability of the results. Furthermore, the absence of objective physiological measurements, such as anorectal manometry, limits interpretations regarding the mechanism of clinical improvement observed after BTI. Nonetheless, the use of long-term follow-up data from a single center, the application of standardized HAEC diagnostic criteria, and the detailed analysis of complications according to surgical techniques are among the strengths of the study.

## 5. Conclusions

Postoperative complications and HAEC remain among the critical challenges determining the clinical course of patients who are undergoing surgery for Hirschsprung’s disease. Although the choice of surgical technique may influence certain complication rates, the development of HAEC appears to be a multifactorial process independent of the surgical approach. Long-segment Hirschsprung’s disease stands out as a prominent risk factor for postoperative complications.

In a selected group of patients with recurrent HAEC episodes and obstructive symptoms, Botulinum toxin injection may be considered a supportive treatment option that could contribute to the management of clinical symptoms. However, due to the retrospective design and limited sample size, it is not possible to draw definitive conclusions regarding the efficacy and long-term outcomes of BTI. Prospective and controlled studies utilizing standardized diagnostic criteria are warranted in this field.

## Figures and Tables

**Table 1:** Delphi Diagnostic Criteria for Hirschsprung-Associated Enterocolitis (HAEC).

Category	Criteria
Clinical Findings	Abdominal distension, explosive diarrhea, fever, lethargy, vomiting, rectal bleeding
Physical Examination Findings	Abdominal tenderness, distension, perianal excoriation, toxic appearance
Radiological Findings	Dilated bowel loops on plain abdominal radiography, air-fluid levels, colonic distension
Laboratory Findings	Leukocytosis or leukopenia, elevated CRP, metabolic acidosis (supportive)
Pathological Findings	Histopathological findings supporting infection or inflammation (if available)
Treatment Requirements	Initiation of antibiotics, need for rectal irrigation, hospitalization
Clinical Course	Response to treatment, history of recurrent episodes

**Table 2:** Number of Patients, Number of Accompanying Anomalies and Syndromes, Types and Number of Surgeries Performed, and Number of Complications that Developed after Surgery.

Total Number of Patients	72	%
Female	12	16.7
Male	60	83.3
Short-segment HD	28	38.9
Long-segment HD	36	50.0
Total Colonic Aganglionosis	8	11.1
Down Syndrome	10	13.8
İmmune Deficiency (Down Syndrome)	8	11.1
Anal Atresia and HD	2	2.7
Crohn Disease	1	1.3
Duodenal Atresia and Non-Rotation Anomaly	1	1.3
Duhamel Procedure	56	77.7
Swenson Procedure	8	11.1
TEPT	8	11.1
Anastomosis Leakage (Swenson)	2	2.7
Anastomosis Leakage (Duhamel)	5	6.9
Anastomosis Leakage (TEPT)	0	0
Anastomosis Stenosis (Swenson)	2	2.7
Anastomosis Stenosis (Duhamel)	10	13.8
Anastomosis Stenosis (TEPT)	0	0
HAEC (Duhamel)	12	16.6
HAEC (Swenson)	2	2.7
HAEC (TEPT)	0	0
BTI Applied	13	18.4
BTI (After a Single Application)	6	8.3
BTI (After Two Times Application)	5	6.9
BTI (Long-Segment HD)	9	12.5
Developing Adhesive Ileus	3	4.1

**Table 3:** Surgical Outcomes, Complication Rates, and BTI Application Success According to Surgical Techniques.

Surgical Techniques	Number of Patients	Anastomosis Leakage (*n*, %)	P (Leakage)	Anastomosis Stenosis (*n*, %)	HAEC (*n*, %)	P (HAEC)	BTI Applied (*n*, %)	BTI Successful (*n*, %)
Duhamel	56	5 (8.9%)	0.209	10 (17.9%)	12 (21.4%)	0.785	13 (23.2%)	11 (84.6%)
Swenson	8	2 (25.0%)		2 (25.0%)	2 (25.0%)		0 (0.0%)	0 (0.0%)
TEPT	8	0 (0.0%)		0 (0.0%)	0 (0.0%)		0 (0.0%)	0 (0.0%)

## Data Availability

The original contributions presented in this study are included in the article. Further inquiries can be directed to the corresponding author.
